# Characterization of *Salmonella* phages isolated from poultry coops and its effect with nisin on food bio‐control

**DOI:** 10.1002/fsn3.3956

**Published:** 2024-01-09

**Authors:** Aysegul Unverdi, Hilal Basak Erol, Banu Kaskatepe, Orkun Babacan

**Affiliations:** ^1^ Department of Pharmaceutical Microbiology Ankara University Faculty of Pharmacy Ankara Turkey; ^2^ Graduate School of Health Science Ankara University Ankara Turkey; ^3^ Department of Veterinary Science, Kepsut Vocational School Balıkesir University Kepsut, Balıkesir Turkey

**Keywords:** food bio‐control, nisin, phage, *Salmonella*

## Abstract

*Salmonella* is a bacterium associated with food contaminated by various animals, primarily poultry. Interest and research on bacteriophages are increasing because they can be used as an alternative against increasing antibiotic resistance. In our study, eight *Salmonella*‐specific lytic bacteriophages were isolated from chicken feces. Two of the isolated phages (AUFM_Sc1 and AUFM_Sc3) were chosen for their characterization due to their broader host range. Based on morphological and genomic analysis, AUFM_Sc1 was identified to be close to similar *Enterobacteria* spp. CC31 (*Myoviridae*) and AUFM_Sc3 was identified to be close to *Salmonella* phage vB_Sen_I1 (*Demerecviridae* (formerly *Siphoviridae*)). Although these phages have shown promise for use in phage therapy applications for chickens, further studies are needed on their suitability. When a cocktail of these phages (AUFM_Sc1 + AUFM_Sc3) and nisin combination was applied on chicken breast meat, it was determined that it was effective against *Salmonella* contamination and while a good inhibitory effect was observed on the food, especially during the first 48 h, the effect decreased later, but the bacterial concentration was still low compared to the control group. Therefore, it is considered that the combination of AUFM_Sc1 + AUFM_Sc3 + nisin can be used as a food preservative against *Salmonella*.

## Graphical abstract

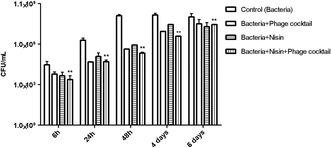

## 1 INTRODUCTION

Foodborne illness and mortality are a major socio‐economic issue, requiring much effort, time and money to identify, treat, and prevent illness. (Crump et al., 2004). *Salmonella*, *Campylobacter*, and *Listeria* are bacteria that are important in foodborne infections, both in public health and in the food industry. Among these, *Salmonella* is one of the most important food safety problems in poultry (Havelaar et al., 2015). It is transmitted to humans and animals by consuming water and food contaminated with *Salmonella*, which is shed from the intestinal tract of rodents, livestock, and humans, especially birds. While *Salmonella* serovars that cause gastroenteritis cause economic losses to producers by delaying the growth of chickens (Remus et al., 2014), they are considered one of the important public health problems by the European Food Safety Authority because they are the most common causes of acute gastroenteritis in humans, the most common of which are *S*. Enteritidis and *S*. Typhimurium serovars (Chowdhury et al., 2023). Therefore, efforts to minimize the prevalence of infection in poultry, which is the primary source for preventing and controlling the *Salmonella* infection worldwide, are important.

To combat *Salmonella* infections, manufacturers prefer to use antibiotics (Clavijo et al., 2022). Inappropriate and excessive use of antimicrobial agents, such as over‐the‐counter and indiscriminate use, is the main factor that causes bacteria to develop resistance to these drugs. However, new resistant variants occur with changes in the genome due to cellular and environmental triggers. These resistance genes can be spread via plasmids. This situation causes the need to develop new antibiotic molecules against new resistant variants. Naturally, the rapid rise of antimicrobial resistance (AMR), called “Silent Epidemic”, is causing worldwide concern. For this reason, although there is a global struggle to reduce the use of antibiotics in humans, efforts are being made to reduce the uncontrolled and high use of antibiotics, especially in veterinary medicine and agriculture, in terms of the One Health Approach. (Durand et al., 2019; Silori et al., 2023). Resistance to several antibiotics (ceftriaxone, norfloxacin, nalidixic acid, chloramphenicol, ciprofloxacin, gentamicin), especially amoxicillin, and resistance to multiple antibiotics has been reported in clinical samples of *Salmonella* spp. (Çiftçi et al., 2019; Erdoğan et al., 2016; Parry & Threlfall, 2008; Terfassa & Jida, 2018). The development of antimicrobial resistance is associated with high number of deaths, prolonged hospitalization, and high costs due to improper treatment. Therefore, the development of resistance to antimicrobials used in *Salmonella* infection is of particular concern for clinical cases (Jajere, 2019).

Data from Republic of Turkiye show that *Salmonella* Enteritidis (57.3%–74.1%) is the most common *Salmonella* serotype in humans, with *Salmonella* Typhimurium (3.0%–8.5%) in second place. *Salmonella* Infantis is the dominant serotype in broiler, turkey, breeder, and slaughterhouse samples, while *Salmonella* Kentucky is the dominant serotype in egg samples. Contamination of *Salmonella* serotype is 47% in slaughterhouse (carcass) samples (Ministry of Agriculture and Forestry, 2018). Resistance to various antibiotics such as ciprofloxacin, ampicillin, tetracycline, trimethoprim‐sulfamethoxazole, and chloramphenicol has been observed in samples of *Salmonella* spp. examined by studies (Çiftçi et al., 2019; Erdoğan et al., 2016; Tural Kara et al., 2015).

The use of bacteriophages, one of the alternatives that can combat against increasingly resistant bacteria and mentioned as a natural control agent, is promising for the control of bacterial infections. Phages, which are viruses that can specifically infect bacteria, can preserve natural microbiota and physicochemical and organoleptic properties while targeting infections in food. Since its first use for therapeutic purposes, phages have been used in areas such as vaccine development, drug applications, development of new antimicrobials against antibiotic‐resistant bacteria, water and food safety, and agriculture and animal health, thanks to the increasing biotechnological developments in recent years (Ashrafudoulla et al., 2021; Fernández et al., 2017; Han et al., 2022; Pujato et al., 2019, Wei et al., 2019).

In the USA, the commercial phage preparations have been approved by the Food and Drug Administration as additives to prevent foodborne illnesses caused by *Listeria monocytogenes* and *Salmonella* spp. (Lang, 2006; Lu et al., 2020). Following the release of the first approved phage product (ListShield™ using for *L. monocytogenes*) many phage products involving different bacteria have been approved for commercial use by various companies and their numbers are increasing. The commercial phage products to control spread of *Salmonella* could be used for human health (*Salmonella* groups A,B,C,D bacteriophage, GastroFag Polyvalent MediPhag, Intestifag® Polyvalent bacteriophage, etc.), animal health (PLSV‐1™, BAFASAL+G®, SalmoFree® etc.) and food contamination (SalmoFresh™, SalmoPro®, PhageGuard S™, etc.) (Huang et al., 2022; Moye et al., 2018). To control *Salmonella*, many *Salmonella* phage studies are still ongoing (Ge et al., 2022; Khan & Rahman, 2022). In addition, as in Pan et al. (2023) study, studies are being carried out that *Salmonella* phages can be used as biocontrol agents on multidrug‐resistant bacteria.

The potential use of phages is recognized as a natural, non‐toxic, viable, and inexpensive technology for the control of *Salmonella* pre‐ and post‐slaughter, especially in poultry (Torres‐Acosta et al., 2019). To determine whether the phage to be used for biocontrol and phage therapy is safe or not, it is necessary to characterize the phage and select the appropriate phage. There are very few studies of the whole genome of phages, which are thought to number more than 10^31^ in nature. More work should be done to expand the existing phage library and to determine the appropriate phage, especially for our country.

In recent years, there has been an increasing trend towards antimicrobials obtained from natural sources as food preservatives. They extend the shelf life of food and can be used to ensure a safe food supply. These compounds are defined by the FDA as Generally Recognized as Safe (Calo et al., 2015). It is not new in the literature to test the efficacy of different combinations of antimicrobials to reduce the antimicrobial concentration used without disturbing the basic structure of food products and to prevent parallel development of resistance (Churklam et al., 2020; Heckler et al., 2021). Nisin is used in the food industry as a safe and natural preservative due to its antimicrobial activity. In particular, nisin is permitted for use in dairy products, processed cheese, creams and is used as a shelf life extender (Balciunas et al., 2013). Therefore, we investigated the combined effect of bacteriophage and nisin against *Salmonella* infection in chicken breast.

An isolated phage is a particularly suitable candidate for a biocontrol agent if it has lytic phage properties (not lysogenic), high phage titer, and wide host range (Chen, Sun, et al., 2018). Therefore, to expand the phage library, more phages should be isolated to ensure the safety of the phage to be used to prevent bacterial contamination (Lu et al., 2020). The aim is to identify the most lytic phage active against *Salmonella* spp., to reveal the characterization of this phage, and to contribute to the literature on the potential and use of phages in food contamination with nisin.

## 2 MATERIALS AND METHODS

### 2.1 Preparation of materials and samples

#### 2.1.1 Bacteria strains and culture conditions

25 *Salmonella* strains, containing 3 *Salmonella* serovars (*S*. Enteritidis, *S*. Typhimirium and *S*. Infantis) obtained from chicken samples in Balikesir, were obtained from Balikesir University Kepsut Vocational School Department of Veterinary Medicine. *Salmonella* spp. strains isolated and identified with biochemical tests, according to ISO 6579, 16 s rRNA gene with PCR and serotyping (Mir et al., 2015). After identified as *Salmonella* spp., PCR was done for identification of *S*. Typhimurium and *S*. Enteritidis using previously defined primers and protocol by Mir et al. (2015). *S*. Infantis was identified by serotyping (ISO 6579). The serotyping was done by According to Kaufmann‐white scheme in accredited reference laboratory, Ankara University Faculty of Veterinary Medicine Microbiology Department. The author thanks to Professor Mehmet Akan for supplied the serotyping. In this study, the *Salmonella* serovars were used as hosts for phages.

#### 2.1.2 Nisin

Nisin‐N5764 (from *Lactobacillus lactis*) was purchased from Sigma‐Aldrich, St Louis, USA. Its 2.5% (v/v) solution was prepared by dissolving in 0.02 N HCl (pH 2.5). It was sterilized by filtration through 0.22 μm membrane filters (Murillo‐Martínez et al., 2013).

#### 2.1.3 Sample collection and processing

Fecal samples were collected from chicken and goose coops in Kahramankazan district of Ankara province Republic of Turkiye, in 2021. Sample processing experiments were performed as described by Phothaworn et al. (2019) with some modifications. Approximately 3 g sample was diluted with 50 mL SM buffer (5.8 g/L of NaCl; 2.0 g/L of MgSO_4_.7H_2_O, 50 mL/L of 1 M Tris–HCl, pH 7.5, 5 mL/L of presterilized 2% gelatin) and completely mixed. Then the mixed liquid was centrifuged at 9000 × *g* for 20 min. The supernatant was filtered into the volumetric flask with a 0.22 μm sterile filter and the filtrate was used in the bacteriophage isolation section.

### 2.2 Methods

#### 2.2.1 Bacteriophage isolation and purification

Mixtures were prepared with 200 μL of each bacterial strain (*S*. Enteritidis, *S*. Typhimirium, 3 *S*. Infantis), 50 mL 2 × TSB (Tyriptic Soy Broth) (Merck, Germany), and 50 mL of the filtrate containing phages. The mixtures were incubated overnight at 37°C with shaking (130 × *g*). The culture was then centrifuged at 9000 × *g* for 20 min repeatedly. The supernatant was filtered through a 0.22 μm sterile filter. The presence of phages in the filtrate content was confirmed by the double layer agar method. Briefly, 200 μL aliquot of the phage filtrate was mixed with 100 μL bacteria selected as the host strain and 3.5 mL TSB added. The mixture was then poured onto the surface of a prepared TSA (Tyriptic Soy Agar) (Merck, Germany) plate and incubated overnight at 37°C. After the presence of the phage plaque was detected, the single plaque was picked using a sterile Pasteur pipette. The important point in this method is to touch the pipette tip to the center of the phage plaque in the petri dish. The phage plaque picked in this way was placed in TSB media. In this process, a single tube was used for each phage plaque. Host bacteria (100 μL) was added. This plaque collection and proliferation process was repeated three times and phage purification was performed (Phothaworn et al., 2019; Sasikala & Srinivasan, 2016).

#### 2.2.2 Host range determination

25 *Salmonella* strains were used for host range determination. The procedure described by Phothaworn et al. (2019) was followed with a change in the processing step. Instead of spotting the phage solution on the bacterial lawn (mixed with bacteria and medium), logarithmic phase *Salmonella* strains, incubated in TSB at 37°C for 3 h before it was used, were inoculated into TSA plate using sterile cotton swabs and phage solutions were spotted on these strips with sterile micropipette. Up to 10 μL phage solutions were dropped onto the strips and incubated overnight. After overnight incubation, a double agar method was performed to control whether the phage had lytic effect on the host and the lytic effect. The expressions (+++, ++, +, −) used for the titles of the lytic activity level of the phage and their explanations are given below (Garbe et al., 2010):

• +++: If the plaque is transparent, there is complete lysis

• ++: If there are very little bacteria

• +: If there is a large amount of bacteria

• −: no lysis if the Petri dish is completely covered with bacteria

#### 2.2.3 The multiplicity of infection (MOI) determination

The effective MOI was calculated by modifying the procedure outlined by Imklin and Nasanit (2020) to obtain a high phage product and show the most efficient pairing of the phage and its host. Briefly, each phage and its host were mixed at 1:1, 1:10, and 1:100. The mixtures were incubated for 4 h at 37°C with shaking (140 × *g*). The cultures were then centrifuged at 9000 × *g* for 20 min and filtered using a 0.22 μm sterile filter. Phage titers were determined by the double agar method.

#### 2.2.4 One‐step growth curve

To determine the life cycle of a virus, the one‐step growth curve was described by Peng et al. (2014) with reference to the procedure. In brief, the phages and their hosts were mixed to obtain the most effective/optimal MOI. The mixtures were incubated at 37°C for 15 min to allow complete absorption. The mixtures were then centrifuged at 15,000 × *g* for 1 min to remove unabsorbed bacteriophages. The pellets were resuspended by gentle vortexing with 1.5 mL of TSB and incubated at 37°C. Then, 100 μL of the phage sample was added every 10 min until the 120th min.

To remove the bacterial structures in each of these samples, they were centrifuged at 15,000 × *g* for 1 min and the supernatants were titrated with free bacteriophage and the phage densities were calculated. The time from which the pellets were first suspended was taken as the start time (zero minutes) of the growth curve. Phage titers were calculated.

#### 2.2.5 The resistance of bacteriophages to environmental conditions

The resistance of the phages (10^6^ CFU/mL) to different pH and temperatures was evaluated. This study was conducted by Jeon et al. (2016) using this method. In order to detect the change in phage titers over a 24‐h period, samples with pH values below 5 and above pH 9 for each phage were tested by keeping them at +4°C. Phage titers were measured after 30 min, 60 min, 90 min, 120 min, 4 h, 6 h and 24 h, respectively. Thermal stability was evaluated by exposure to 50°C and 70°C for 1, 2, 4 and 6 h, respectively. Control phage samples were maintained at pH 7.5 and stored at +4°C.

#### 2.2.6 Morphological phage characterization

Approximately 10 μL of purified phage particles were added onto a formvar/carbon‐coated grid and negatively stained with 0.5% uranyl assay. The morphological images of the phages were obtained with a transmission electron microscope (Hitachi HT7800) at a voltage of 80 kV in the Faculty of Medicine Histology‐Embryology Department at Ankara University.

#### 2.2.7 Whole genome sequencing

DNA isolation from phage samples was performed by BMLabosis company, Ankara, Republic of Turkiye. Bioinformatics studies were carried out at Ankara University Technopolis AgriGenomics Hub Animal and Plant Genomics Research Innovation Center. Here, Illumina adapter sequences were trimmed before assembling raw fast files with a novo assembler using Geneious Prime, and low‐quality reads (<Q30) were filtered using Geneious Prime version 2022.2.1. The alignment of forward and reverse sequencing files and paired ends were combined and then trimmed to Phred scores more than 30 performed using BBDuk. Genome annotation was performed by analysis of the obtained fragments (contig) using BLASTP. The BLASTN program was used to identify the genome‐wide closest relatives of the phage. Molecular identifications were completed by evaluating 10 genomes with the highest Bit score and highest pairwise identity.

#### 2.2.8 The combine effect of phage cocktail and nisin on food biocontrol

According to host range analysis, two phages (AUFM_Sc1, AUFM_Sc3) with high lytic activity were selected for food biocontrol studies. The effect of nisin and phage cocktail (alone and in combination) on food biocontrol against *S*. Infantis was also investigated according to Bao et al. (2015) method with time modification. In our study, the effects of nisin and phage on contamination over longer periods of time were investigated. Chicken breast was purchased from a local grocery store. Chicken meat, which was brought to the laboratory with its packaging intact, was cut into 2 × 2 cm pieces with a sterile cutter under aseptic conditions. Each piece of chicken meat was added to sterile petri dishes. A suspension of bacteria was prepared at 0.5 MacFarland turbidity in overnight fresh bacterial culture and the final concentration was adjusted to 5 × 10^5^ CFU/mL. 100 μL of the bacterial suspension was added to each chicken meat and allowed to stand for 15 min. The AUFM_Sc1 and AUFM_Sc3 phages were then mixed in equal proportions to form a phage cocktail of 10^7^ PFU/mL. The chicken meat was divided into four groups. 100 μL of phage cocktail or nisin and 50 μL nisin+50 μL phage cocktail were added to the groups. As a control, an equal volume of PBS was added instead of antibacterial agent. Chicken meats in all groups were kept at 4°C. At the end of the 6 h, 24 h, 48 h, 4th day and 6th day incubation periods, a piece of chicken meat sample was taken and added to 5 mL of 0.85 NaCl solution and incubated for 10 min at room temperature. It was then vortexed for 1 min and centrifuged at 3000 × *g* for 10 min. The supernatant was discarded and suspended in the pellet with 1 mL of PBS, then diluted with PBS and the dilutions were spread on the TSA plate and incubated 37°C for enumeration of viable counts.

#### 2.2.9 Statistical analysis

Each experiment was repeated three times. The results were presented as mean values and standard deviation values of the mean. Friedman test (*p* < .05; GraphPad Prism version 5) was used to determine statistically significant differences between the treatment and control groups.

## 3 RESULTS

### 3.1 Bacteriophage isolation and purification

Obtained from chicken feces samples used in the study, 8 phages (AUFM_Sc1, AUFM_Sc2, AUFM_Sc3, AUFM_Sc4, AUFM_Sc5, AUFM_Sc6, AUFM_Sc7, AUFM_Sc8) were isolated from chicken feces. Plaques of AUFM_Sc4 and AUFM_Sc6 phages were larger than plaques of other isolated phages. The AUFM_Sc7 phage plaque was the least numerous. In addition, the plaques of the AUFM_Sc3 and AUFM_Sc7 phages have smaller plaques than those isolated, and the plaques were difficult to detect. According to the general morphology of the lytic plaques of these phages, one or two plaques were selected from each Petri dish, and phage purification was carried out by a single plaque collection method.

### 3.2 Host range determination

It is important to determine the host range to select the most suitable phage as biocontrol agent. For this purpose, the Spot Test method was followed with 25 strains containing three *Salmonella* serovars (*S*. Enteritidis, *S*. Typhimurium and *S*. Infantis) of eight isolated phages (Table S1). Based on the percentage of lytic activity AUFM_Sc1 and AUFM_Sc3 phages were found to have the highest percentage of lytic activity, 84% and 92%, respectively. Phage AUFM_Sc3 was the phage with the highest activity. Since the plaque morphology of phage AUFM_Sc1 was more opaque and wider than that of phage AUFM_Sc3. This study continued with phages AUFM_Sc1 and AUFM_Sc3.

### 3.3 Multiplicity of infection (MOI), One‐Step growth curve and resistance to environmental conditions

**FIGURE 1:**
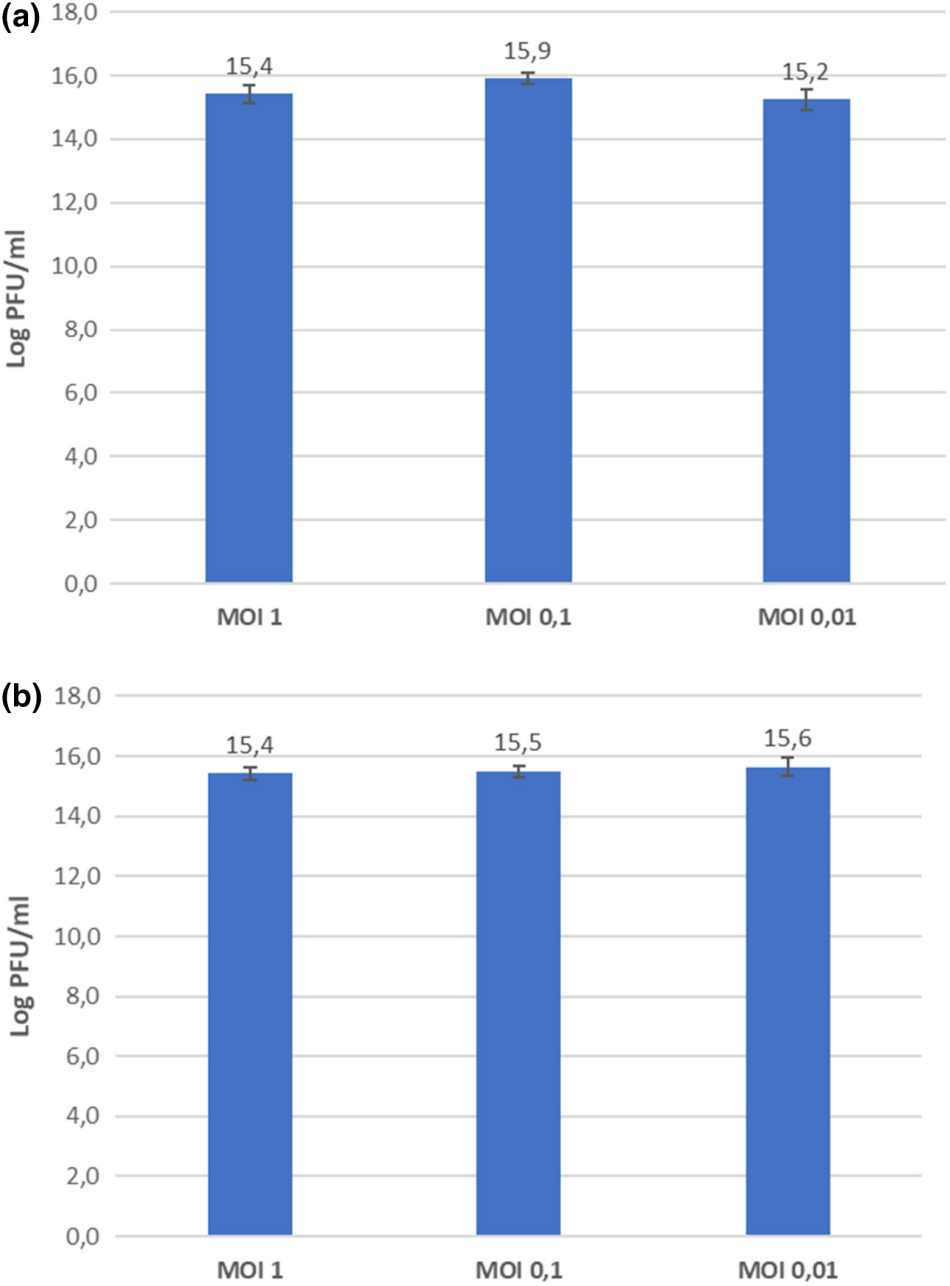
The optimal MOI values of phages AUFM_Sc1 (a) and AUFM_Sc3 (b). All values provided are expressed as mean ± standard deviation in triplicate.

**FIGURE 2:**
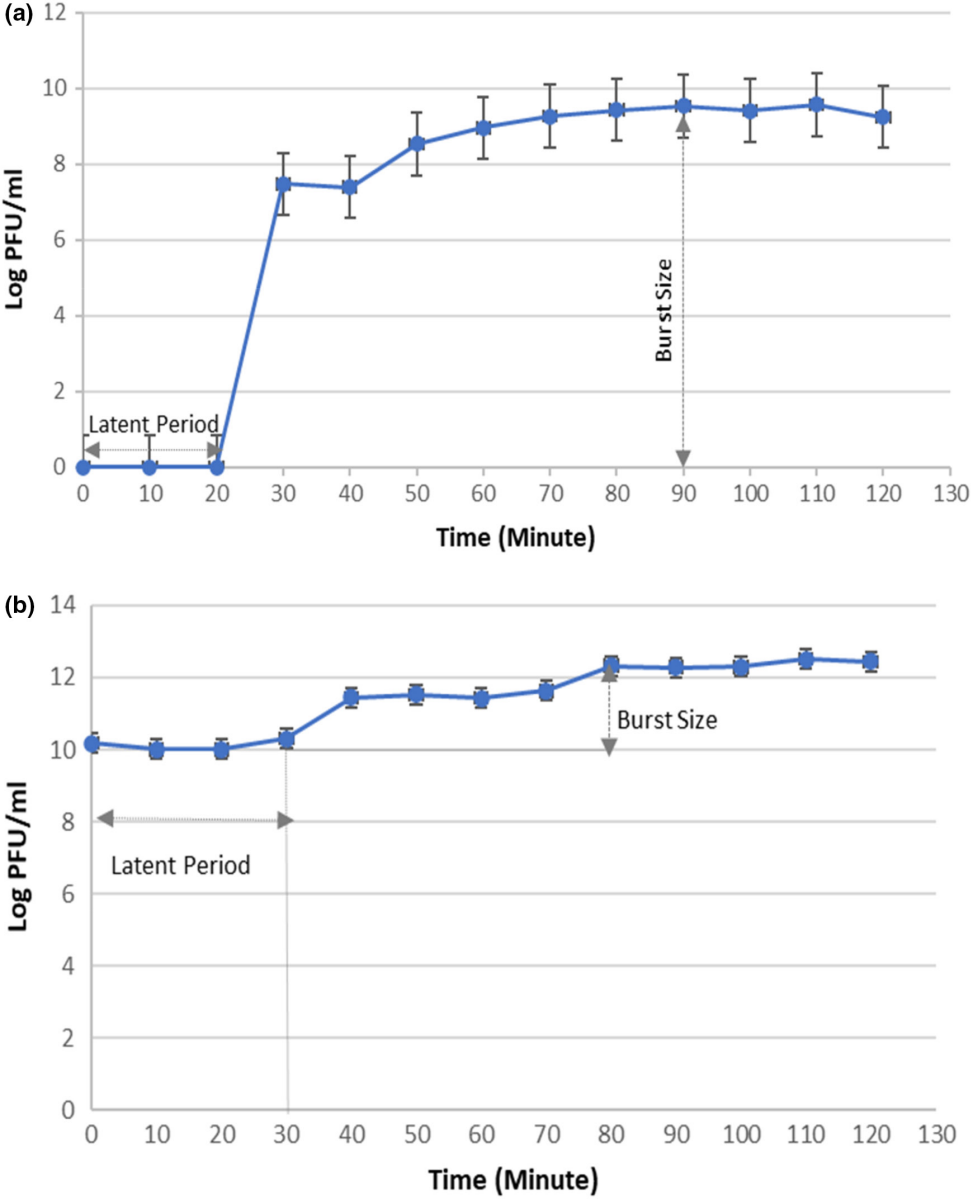
One step growth curve of phages AUFM_Sc1 (a) and AUFM_Sc3 (b). All values provided are expressed as mean ± standard deviation in triplicate.

The optimal MOI values can be different depending on the number of infecting phages, the rate of attachment to the host cell, the attachment time, and the density of the host. According to the MOI test result (Figure 1), the optimal MOI values of phages AUFM_Sc1 and AUFM_Sc3 are 0.1 and 0.01, respectively. The one‐step growth curve showed that phages AUFM_Sc1 and AUFM_Sc3 had latent periods of 20 and 30 min, respectively. Furthermore, the burst size of phages AUFM_Sc1 and AUFM_Sc3 was 342 PFU/mL and 212 PFU/mL, respectively (Figure 2). Since these values are host‐dependent, the results can alter with a different *Salmonella* host strain.

**FIGURE 3:**
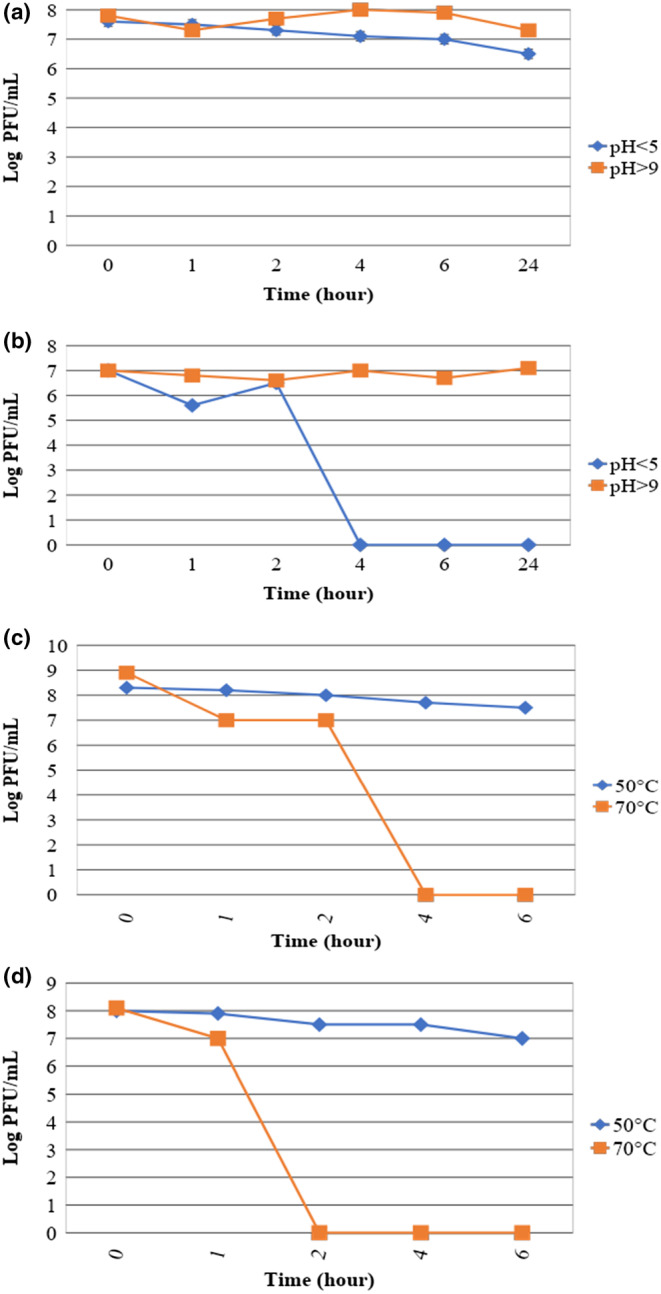
The pH stability of phages AUFM_Sc1 (a), AUFM_Sc3 (b); the thermal stability of phages AUFM_Sc1 (c), AUFM_Sc3 (d).

Environmental resistance of the phages was determined as shown in Figure 3. Phage AUFM_Sc1 was found to be more resistant to particularly low pH than phage AUFM_Sc3. It was observed that phage AUFM_Sc3 could not show resistance after 2 h in low pH environment. It was observed that both phages maintained their activity in environments above pH 9, and there was no significant change in phage titers. The changes in phage titers over 6 h at temperatures between 50°C and 70°C were evaluated. A slight decrease in phage AUFM_Sc1 observed over time at temperatures of 50°C for both phages AUFM_Sc1 and AUFM_Sc3. However, this decrease is not significant. At temperatures of 70°C, the activities of phages AUFM_Sc1 and AUFM_Sc3 were lost after 2 and 1 h, respectively.

### 3.4 Phage morphology

**FIGURE 4:**
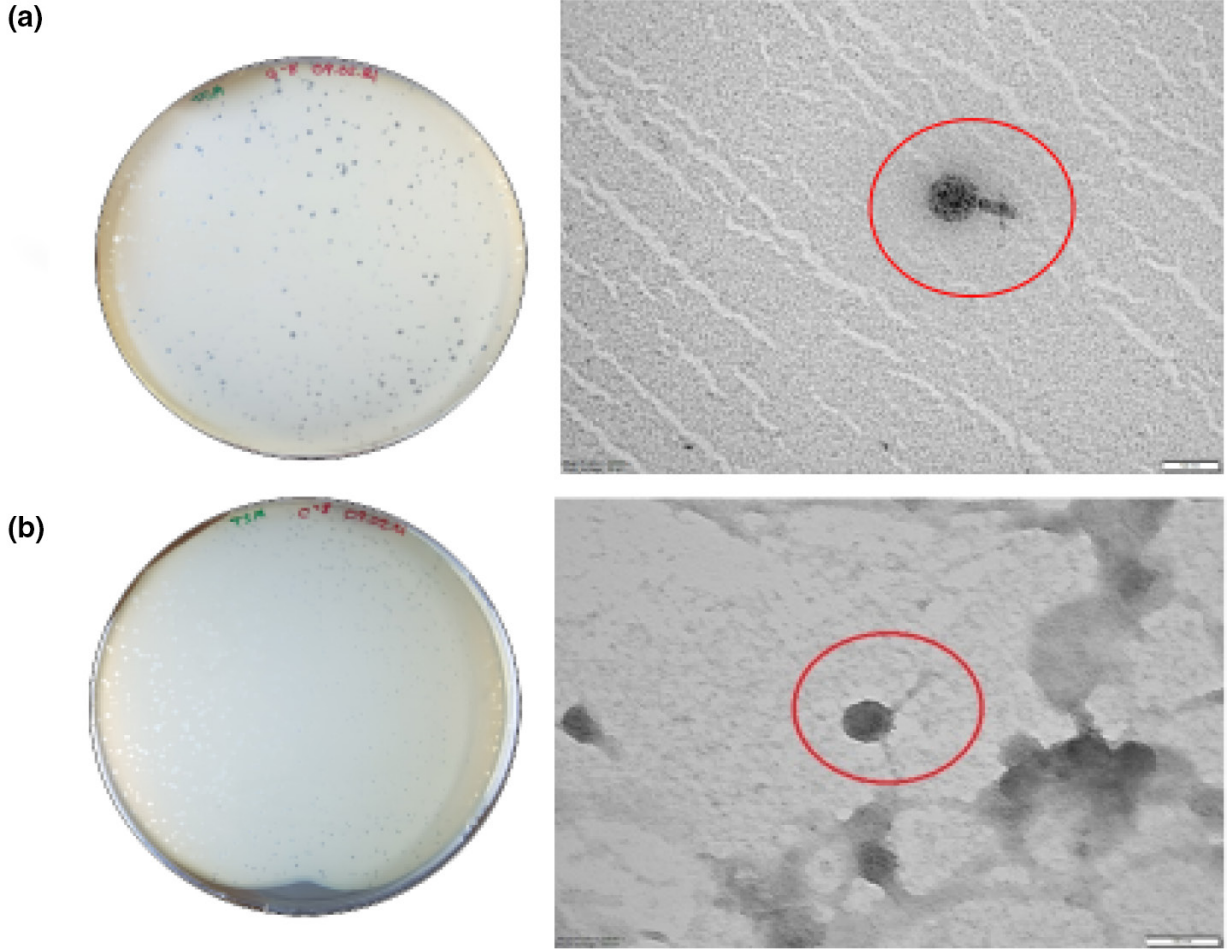
Plaque morphology and TEM images of AUFM_Sc1 (a) and AUFM_Sc3 (b).

The selected and purified phages were morphologically classified using transmission electron microscopy (TEM) as shown in Figure 4. The micrographs revealed that AUFM_Sc1 phage has morphology of members of the *Myoviridae* family with a typical icosahedral head and a short contractile tail, and AUFM_Sc3 phage has morphology of members of the *Demerecviridae* (formerly *Siphoviridae*) family with an icosahedral head and a long noncontractile tail. The approximate diameter of the AUFM_Sc1 phage head was 55.5 nm, and the tail length was 55.5 nm, and the AUFM_Sc3 phage head was 31.6 nm and the tail length was 88.8 nm.

### 3.5 Phylogenetic analysis

A comparison with the NCBI data on isolated phages shows that the highest homology at the nucleotide level is *Enterobacteria* phage CC31 (Accession numb. GU323318.1) with a 92.9% pairwise identity for the AUFM_Sc1 phage and *Salmonella* phage vB_Sen_I1 (Accession numb. MT233524.1) with a pairwise identity of 93.1% for the AUFM_Sc3 phage (Table S2).

### 3.6 The effect of phage cocktail and nisin

**FIGURE 5:**
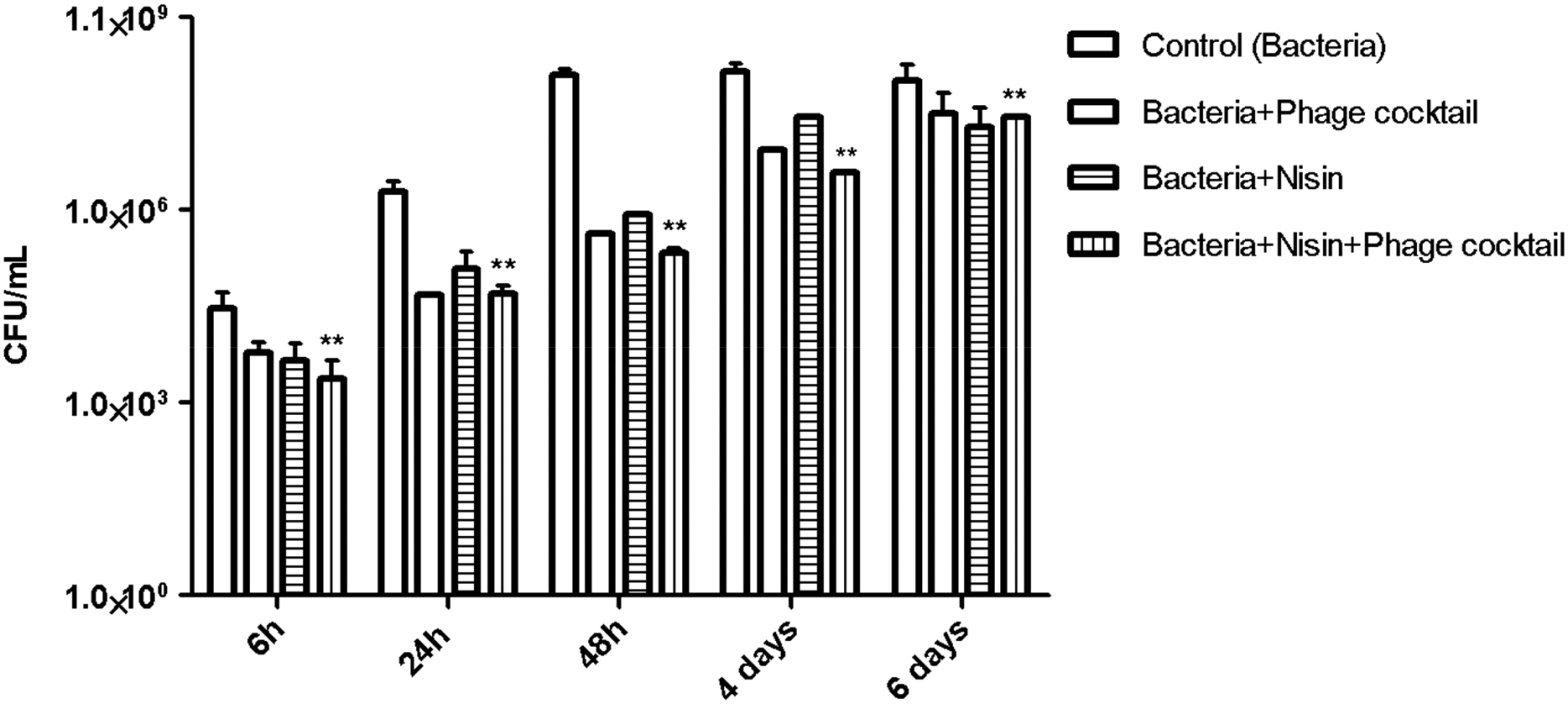
Effect of phage cocktail (AUFM_Sc1 + AUFM_Sc3) and nisin against *Salmonella* contamination on the surface of chicken breast. Data represent the mean ± S.D. (*n* = 3), ** represents *p* < .05 (Friedman test) significant difference observed when compared with the controls (in the absence of phage).

The antibacterial activity of phage cocktail, nisin, and their combination against food contamination with *Salmonella* is shown in Figure 5. It can be seen that nisin and phage cocktail applications have similar efficacy levels in the first 6 h. Despite the rapid increase in bacteria in the control, phage cocktail and nisin applications maintain their efficacy in the first 48 h. However, on days 4 and 6 days, there is a 0.5 log CFU/mL decrease in the bacterial count with the combined application compared to the control. The combined effect of nisin and phage cocktail was observed throughout the incubation period. Bacterial regrowth was not seen in the nisin+phage cocktail treatment at 48 h. Although the combined treatment of nisin and phage cocktail showed a small combined effect, it was still more effective than the single treatments, reducing viable counts by ~2 log_10_ logs at the end of the incubation period.

## 4 DISCUSSION

Efforts are being made worldwide to ensure the safety of finished products such as raw chicken. However, some approaches (UV light, irradiation etc.) are insufficient for pathogens that cause poultry‐contaminated infections and pathogenic microorganisms (Chowdhury et al., 2023). Antibiotics used for infections not only cause bacteria to gain resistance, but also cause disruption of the animal's natural flora. Compared to antibiotics, bacteriophages are specific for certain bacteria, infecting only one species, serotype, or strain, so they do not cause destruction of the common intestinal flora. Additionally, self‐replication of bacteriophages occurs during treatment, eliminating the need for their repeated administration (Loc‐Carrillo & Abedon, 2011) Further, it is predicted that bacteriophages can be used not only to reduce bacterial contamination in animals, but also as safe disinfectants in industry to reduce contamination on food contact surfaces or poultry carcasses (Abd‐El Wahab et al., 2023). It is stated that phage therapy may be helpful in reducing horizontal transmission of *Salmonella* in poultry (Wernicki et al., 2017). Phage therapy has some limitations, such as the need for sufficient phage titer and their ability to survive in the chicken caeca for a sufficient period after the therapy period. Therefore, the choice of phage used in phage therapy is very important. Newly isolated BPSELC‐1 and BPSELC‐6 phages could lyse 26 *Salmonella* serotypes, including the important pathogens *S*. Enteritidis and *S*. Typhimurium and *S*. Pullorum (Li et al., 2020).

In this study that was aimed to isolate new *Salmonella* phages and identify the appropriate phage that can be used for phage therapy and/or biocontrol agents against *Salmonella* in poultry. Among eight phages were isolated from chicken feces in this study, two selected phages (AUFM_Sc1 and AUFM_Sc3) showed high lytic activity against three hosts (*S*. Enteritidis, *S*. Typhimurium, and *S*. Infantis).

The test performed to determine the effective titer ratio of phage to the bacterial host revealed that the MOI value of the AUFM_Sc1 phage was 0.1 while the MOI value of the AUFM_Sc3 phage was 0.01. The high MOI of AUFM_Sc1 indicates that it is appropriate to use high phage concentration to reduce the bacterial population. This means that the AUFM_Sc1 phage has a higher titer than the AUFM_Sc3 phage to destroy the *Salmonella* host cell (Chen, Yuan, et al., 2018). It is considered that it would not be appropriate to use the phage as biocontrol agents just by looking at its high MOI value (Abedon, 2016).

Foods are biochemically digested by pepsin in the stomach, which is the region with the lowest pH (1.5–3.5) in chickens (Tabata et al., 2017), and the passage of these nutrients through the gastrointestinal tract takes about 5 h (Gauthier, 2002). Considering the acid stability of the studied phages in the environment, when the phages are considered, it was found that the AUFM_Sc1 phage is more resistant than the AUFM_Sc3 phage, especially against values lower than pH 5. It was observed that in environments where the pH of AUFM_Sc1 phage is lower than 5, its titer can be relatively sufficient to infect bacteria. Although the AUFM_Sc3 phage appears to be more sensitive to low pH, it has been determined that phage activity persists for at least 2 h. In this case, it is considered that it can leave the stomach with a low pH level without losing most of its titer. In the thermal endurance tests of the AUFM_Sc1 and AUFM_Sc3 phages obtained in this study, it was observed that both phages show activity on bacteria at temperatures of 50°C. Considering that the body temperature of poultry is between 41 and 42°C, AUFM_Sc1 and AUFM_Sc3 phages have sufficient thermal endurance for oral phage therapy.

The one‐step growth curve shows the correlation between the logarithm of phage titer and infection time. Studies have reported that the latent period for *Salmonella* phages is between 15–30 min. Sritha and Bhat (2018) stated that the latent period of the isolated ΦStp1 phage was 30 min and the burst size was 37 PFU/mL. In their study, Duc et al. (2018) showed that the latent period of 5 phage isolates was 15–25 min, and the burst size was approximately 49–209 PFU/mL. In our study, the latent period of the characterized phage AUFM_Sc1 was 20 min, and that of phage AUFM_Sc3 was determined to be 30 min. It can be seen that the phages obtained in our study show similar results to those reported in literature.

Transmission electron microscopy and genome analysis are frequently used to identify bacteriophages. There are some difficulties in comparing the studied phage genomes with each other. For example, if two phages infecting the same host are not closely related or a close prophage of the other, it is unusual to find nucleotide similarity between these phages. The genome mosaic that results from horizontal genetic variation, which is more common in phages than in bacteria, also complicates genome comparison between phages. In addition, the frequency of recombination is high due to extranuclear genes that are rapidly located and separated in the phage genome in a short time (Hatfull & Hendrix, 2011). In studies in the literature, it is seen that phages infecting *Salmonella* bacteria are generally in the families *Demerecviridae* (formerly *Siphoviridae*) and *Myoviridae* (Gao et al., 2023; Li et al., 2020; Olsen et al., 2022; Wang et al., 2023). When the phages we isolated in this study were compared with the data in NCBI, it was observed that they were quite different in terms of genomics, even though they infected the same host. Because of the genetic analysis of the AUFM_Sc3 phage, it was observed that the AUFM_Sc3 phage belongs to the *Demerecviridae* family and has similarities with *Escherichia* virus T5, *Escherichia* phage slur09, and *Salmonella* phage vB_Sen_I1 phages. On the other hand, phage AUFM_Sc1 belongs to the Myoviridae family and is similar to *Enterobacteria* phage CC31.

The phages vB_SenS_CSP01, vB_Sens_PHB06, and vB_SenS_PHB07, which belong to the *Demerecviridae* family were isolated by Chen, Sun, et al. (2018), inhibited the growth of *S*. Enteritidis and reduced the biofilm of *S*. Typhimurium at pH (3–11), and temperature (4–50) changes. Therefore, it is predicted that these phages can be used as biocontrol agents. Phages obtained from chicken feces (ST‐W77 phage, *Myoviridae*, and SE‐W109 phage, *Demerecviridae*) may be suitable for use as a biocontrol agent that can destroy *Salmonella* not only on chicken meats but also on milk and dairy products (Phothaworn et al., 2020). A cocktail containing 6 *Salmonella* lytic phages was investigated to determine the efficacy of SalmoFREE® and the control of *Salmonella* contamination in poultry products (Clavijo et al., 2019). At the end of this study, it was concluded that these phages can be successfully applied without adverse effects on chickens in a commercial broiler farm.

The studies conducted in the Turkiye, have shown that the phages obtained can be effective against the *Salmonella* pathogen. In the study of Demiraslan Aydin (2018), one of them, 4 phages (SEnt‐F1, F2, F3, and F4) obtained from chicken meat and skin were more effective against *S*. Enteritidis MET‐S1‐411, especially when applied as a cocktail, and similarly to the family *Demerecviridae*. Urgaci (2019) has shown that SEnt‐P5 and SEnt‐P6 phages of the genus *S*. Enteritidis has the potential to be used against the foodborne pathogen *S*. Enteritidis. it was emphasized that *Salmonella* phages (*Myoviridae*: PSCs1, PSDs1, PSSr1 and PSMc1; *Demerecviridae*: PSCs2) and commercial phages (Salmonellex™ phage) should be used to prepare cocktails containing *Salmonella* phages against antibiotic‐resistant *Salmonella* spp. and biofilms (Heshmati, 2019). It was reported that *Salmonella*‐specific BS19 phage may be suitable for use in biocontrol applications due to its stable infection capacity even under different environmental conditions obtained from village poultry houses (Akpinar, 2020).

It is recommended to use phages as a phage cocktail rather than monophages with the phage cocktail, as it can provide the elimination of *Salmonella* bacteria at lower doses. For this reason, it is considered appropriate to use the phages obtained in phage cocktails, considering their current properties (Gu et al., 2012). Although it is general believed that phages don't disrupt the natural microbiota, they are safe. After identifying the phage/phages that may be suitable for phage therapy, studies should be conducted to increase the reliability of the phage by conducting studies on whether the phage to be used will have an effect on the patient/normal individual. Curious about the effects of phage therapy on the natural microbiota, Clavijo et al. (2022) found that although there was no effect of the commercial product SalmoFree phage cocktail on the growth of normal microbiota there was an increase in some bacteria present. For this reason, it is recommended to investigate that the effects on the individual microbiota be assessed each phage application. Based on the research, it is recommended to test the reliability of the application by preparing a simulated environment before the application of phages, that are predicted to be suitable as phage therapy, and to administer the phages that are successful in the simulation to the live poultry and to monitor and record the results. A model that could be constructed using synthetic biology techniques could ensure the protein stability of the phage to generate variants that are both safe and stable (Pelyuntha & Vongkamjan, 2022; Strobel et al., 2022).

There are studies investigating the efficacy of nisin with other natural preservatives against pathogens. Heckler et al. (2021) investigated the combined effect of carvacrol, thymol, and nisin against *S. aureus* and *Salmonella* and found that it caused a reduction of 1.2 log CFU/mL for MIC and 4.98 log CFU/mL for 2MIC for *S. aureus*. Abarca et al. (2022) produced a biodegradable film from a mixture of nisin and EDTA and reported reduction of 3 log_10_ for *E. coli*. In addition to this study, it was founded only one study about nisin phage combination in literature. Duc et al. (2023) isolated STG2, SEG5, and PS5 phages against *S. typhimurium*, *S. enteritidis*, and *E. coli* O157:H7 responsible for food poisoning. They reported that when they combined the phage cocktail they prepared from these phages with nisin and EDTA, the antibacterial effect on the bacterial mixture increased and the phage resistance decreased.

## 5 CONCLUSIONS

It has been observed that the combined application of bacteriophage and nisin, which we offer as a natural solution to food contamination, has an increasing effect on food safety, and it has been found to preserve the food within 48 h, especially in the case of possible contamination. However, unfortunately, the number of bacteria could not be reduced below the detection limit. The study should be enriched by searching for the existence of phage‐resistant bacterial mutants.

## AUTHOR CONTRIBUTIONS

**Aysegul Unverdi:** Conceptualization (equal); formal analysis (equal); investigation (equal); methodology (equal); visualization (equal); writing – original draft (equal). **Hilal Basak Erol:** Conceptualization (equal); formal analysis (equal); methodology (equal); visualization (equal); writing – original draft (equal). **Banu Kaskatepe:** Conceptualization (equal); formal analysis (equal); funding acquisition (equal); investigation (equal); methodology (equal); visualization (equal); writing – review and editing (equal). **Orkun Babacan:** Formal analysis (equal); methodology (equal).

## FUNDING INFORMATION

This work was supported by the Ankara University Scientific Research Projects Coordination Unit [grant number 21 L0237004].

## CONFLICT OF INTEREST STATEMENT

The authors declare no conflict of interest.

## ETHICS STATEMENT

This declaration is not applicable, as no clinical study was performed in the current study.

## Supporting information


Table S1.
Table S2.

## Data Availability

The authors confirm that the data supporting the findings of this study are available within the article.
