# Supplementary material for: Characterization of Salmonella phages isolated from poultry coops and its effect with nisin on food bio‐control
Source: Food Sci Nutr. 2024 Jan 9;12(4):2760–71. doi: 10.1002/fsn3.3956 (PMC11016409; doi:10.1002/fsn3.3956)
Supplement: Supplementary file 1 — Table S1. Table S2. [file FSN3-12-2760-s001.docx]

# **Characterization of *Salmonella* phages isolated from poultry coops and its effect with nisin on food bio-control**

Aysegul UNVERDI^1,2^, Hilal Basak EROL^1^, Banu KASKATEPE^1*^, Orkun BABACAN^3^

^1^Ankara University Faculty of Pharmacy, Department of Pharmaceutical Microbiology, Ankara/Turkey

^2^ Ankara University Graduate School of Health Science, Ankara, Turkey

^3^Department of Veterinary Science, Kepsut Vocational School, Balıkesir University, Kepsut, Balıkesir, Turkey

^*^Corresponding author: Banu KASKATEPE, email: [bkaskatepe@ankara.edu.tr](mailto:bkaskatepe@ankara.edu.tr)

**Supplementary Table 1.** Host range analysis of novel isolated *Salmonella* phages

|  | AUFM_Sc1 | AUFM_Sc2 | AUFM_Sc3 | AUFM_Sc4 | AUFM_Sc5 | AUFM_Sc6 | AUFM_Sc7 | AUFM_Sc8 |
| --- | --- | --- | --- | --- | --- | --- | --- | --- |
|  |  |  |  |  |  |  |  |  |
| Si 1 | **++** | **+** | **+++** | **+** | **+** | **+** | **-** | **+** |
| Si 3 | **+++** | **++** | **+++** | **+** | **+** | **+** | **-** | **+** |
| Si 4 | **+++** | **+++** | **+++** | **+** | **+** | **+** | **-** | **-** |
| Si 5 | **+++** | **+++** | **+++** | **+** | **+** | **+** | **-** | **++** |
| Si 8 | **+** | **+** | **+++** | **+** | **+** | **+** | **-** | **+** |
| Si 10 | **+++** | **+++** | **+++** | **+** | **+** | **+** | **-** | **+** |
| Si 11 | **+++** | **+** | **+++** | **+** | **+** | **+** | **-** | **+** |
| Se 12 | **+++** | **+++** | **+++** | **+** | **+** | **++** | **-** | **-** |
| Si 14 | **+++** | **++** | **+++** | **+** | **+** | **+** | **-** | **+** |
| Si 15 | **+++** | **+** | **+++** | **+** | **+** | **+** | **-** | **+** |
| Si 17 | **+++** | **+** | **-** | **-** | **-** | **-** | **-** | **-** |
| Si 21 | **+++** | **+++** | **+++** | **+** | **+** | **+** | **-** | **+** |
| Si 26 | **+++** | **++** | **+++** | **+** | **+** | **+** | **-** | **++** |
| Si 27 | **+++** | **+++** | **+++** | **+** | **+** | **+** | **-** | **+** |
| Si 29 | **+++** | **+++** | **+++** | **+** | **+** | **+** | **-** | **+** |
| Si 30 | **++** | **+++** | **-** | **-** | **-** | **?+** | **-** | **-** |
| Si 31 | **++** | **+++** | **+++** | **+** | **+** | **+** | **-** | **+** |
| Si 33 | **++** | **+++** | **+++** | **+** | **+** | **+** | **-** | **+** |
| Si 34 | **+** | **+** | **+++** | **+** | **+** | **+** | **-** | **+** |
| Si 38 | **+** | **+** | **+++** | **+** | **+** | **+** | **-** | **++** |
| Si 39 | **+** | **+** | **+++** | **+** | **+** | **+** | **-** | **+** |
| Si 42 | **+++** | **+++** | **+++** | **+** | **+** | **+** | **-** | **+** |
| Si 44 | **++** | **++** | **+++** | **+** | **+** | **+** | **-** | **+** |
| Si 47 | **+++** | **+++** | **+++** | **+** | **+** | **+** | **-** | **++** |
| St 48 | **+++** | **+** | **+++** | **+** | **+** | **+** | **-** | **++** |
| % of lytic activity | **84%** | **64%** | **92%** | **0%** | **0%** | **4%** | **0%** | **20%** |

**Si:*Salmonella* Infantis**

**Se: *Salmonella* Enteritidis**

**St: *Salmonella* Typhimirium**

**Supplementary Table 2.** Phylogenetic analysis of AUFM_Sc1 (A) and AUFM_Sc3 (B) phages.

A

| **Similarity rate** | **Accession number** | **Bit-score** | **Description** | **Sequence Length** | **Taxonomy** |
| --- | --- | --- | --- | --- | --- |
| 92.9% | GU323318.1 | 183,939 | Enterobacteria phage CC31 | 165540 | Viruses: Duplodnaviria, Heunggongvirae, Uroviricota, Caudoviricetes, Caudovirales, Myoviridae, Tevenvirinae, Karamvirus |
| 98.9% | MN087708.1 | 169,165 | Enterobacter phage vB_EhoM-IME523 | 172763 | Viruses: Duplodnaviria; Heunggongvirae; Uroviricota; Caudoviricetes; Caudovirales; Myoviridae; Kanagawavirus; unclassified Kanagawavirus |
| 100.0% | NC_055726.1 | 167,319 | Cronobacter phage Pet-CM3-4 genome assembly | 171975 | Viruses: Duplodnaviria, Heunggongvirae, Uroviricota, Caudoviricetes, Caudovirales, Myoviridae, Tevenvirinae, Karamvirus, unclassified Karamvirus |
| 97.9% | OL989991.1 | 165,472 | Enterobacter phage vB_EclM_Q7622 | 173871 | Viruses: Duplodnaviria; Heunggongvirae; Uroviricota; Caudoviricetes; Caudovirales; Myoviridae; Kanagawavirus; unclassified Kanagawavirus |
| 100.0% | OL828291.1 | 165,472 | Enterobacter phage vB-EclM_KMB20 | 174428 | Viruses: Duplodnaviria, Heunggongvirae, Uroviricota, Caudoviricetes, Caudovirales, Myoviridae, Tevenvirinae, Karamvirus, unclassified Karamvirus |
| 100.0% | OL828290.1 | 165,472 | Enterobacter phage vB-EclM_KMB19 | 172697 | Viruses: Duplodnaviria, Heunggongvirae, Uroviricota, Caudoviricetes, Caudovirales, Myoviridae, Tevenvirinae, Karamvirus, unclassified Karamvirus |
| 100.0% | OK210076.1 | 165,472 | Enterobacter phage KKP_3262 | 84075 | Viruses: Duplodnaviria, Heunggongvirae, Uroviricota, Caudoviricetes, Caudovirales, Myoviridae, Tevenvirinae, Karamvirus, unclassified Karamvirus |
| 100.0% | NC_055739.1 | 165,472 | Enterobacter phage myPSH1140 | 172614 | Viruses: Duplodnaviria, Heunggongvirae, Uroviricota, Caudoviricetes, Caudovirales, Myoviridae, Tevenvirinae, Karamvirus, unclassified Karamvirus |
| 100.0% | MN013084.1 | 165,472 | Klebsiella phage vB_KaeM_KaAlpha | 172334 | Viruses: Duplodnaviria, Heunggongvirae, Uroviricota, Caudoviricetes, Caudovirales, Myoviridae, Tevenvirinae, Karamvirus |
| 100.0% | KJ101592.1 | 165,472 | Enterobacter phage PG7 | 173276 | Viruses: Duplodnaviria, Heunggongvirae, Uroviricota, Caudoviricetes, Caudovirales, Myoviridae, Tevenvirinae, Karamvirus |

B

| **Similarity rate** | **Accession number** | **Bit-score** | **Description** | **Sequence Length** | **Taxonomy** |
| --- | --- | --- | --- | --- | --- |
| 93.1% | MT233524.1 | 28258,6 | Salmonella phage vB_Sen_I1 | 112111 | Viruses: Duplodnaviria, Heunggongvirae, Uroviricota, Caudoviricetes, Caudovirales, Demerecviridae, Markadamsvirinae, Tequintavirus, Unclassified Tequintavirus |
| 91.6% | NC_047859.1 | 27156,1 | Salmonella phage SP01 | 117842 | Viruses: Duplodnaviria, Heunggongvirae, Uroviricota, Caudoviricetes, Caudovirales, Demerecviridae, Markadamsvirinae, Tequintavirus, Tequintavirus SP01 |
| 91.7% | MW006479.1 | 22526,6 | Salmonella phage GEC_vB_N5 | 110015 | Viruses: Duplodnaviria, Heunggongvirae, Uroviricota, Caudoviricetes, Caudovirales, Demerecviridae, Markadamsvirinae, Tequintavirus, Unclassified Tequintavirus |
| 87.5% | NC_047754.1 | 20992 | Escherichia phage APCEc03 | 103737 | Viruses: Duplodnaviria, Heunggongvirae, Uroviricota, Caudoviricetes, Caudovirales, Demerecviridae, Markadamsvirinae, Tequintavirus, Tequintavirus APCEc03 |
| 87.0% | MW149273.1 | 20493,4 | Salmonella phage vB_SalS_ABTNLsp4 | 106178 | Viruses: Duplodnaviria, Heunggongvirae, Uroviricota, Caudoviricetes, Caudovirales, Demerecviridae, Markadamsvirinae, Tequintavirus, Unclassified Tequintavirus |
| 86.6% | NC_048748.1 | 20101,9 | Escherichia phage vB_EcoS_HdH2 | 120120 | Viruses: Duplodnaviria, Heunggongvirae, Uroviricota, Caudoviricetes, Caudovirales, Demerecviridae, Markadamsvirinae, Tequintavirus, Tequintavirus HdH2 |
| 86.4% | ON185583.1 | 19686,4 | Escherichia phage EC122 | 108723 | Viruses: Duplodnaviria, Heunggongvirae, Uroviricota, Caudoviricetes, Caudovirales, Demerecviridae, Markadamsvirinae, Tequintavirus, Unclassified Tequintavirus |
| 94.5% | LN887948.1 | 18151,8 | Escherichia phage slur09 | 111751 | Viruses: Duplodnaviria, Heunggongvirae, Uroviricota, Caudoviricetes, Caudovirales, Demerecviridae, Markadamsvirinae, Tequintavirus |
| 93.9% | MT843274.1 | 17799,1 | Escherichia phage BB1 | 110099 | Viruses: Duplodnaviria, Heunggongvirae, Uroviricota, Caudoviricetes, Caudovirales, Demerecviridae, Markadamsvirinae, Tequintavirus, Unclassified Tequintavirus |
| 85.6% | ON185584.1 | 16984,8 | Escherichia phage EC142 | 108723 | Viruses: Duplodnaviria, Heunggongvirae, Uroviricota, Caudoviricetes, Caudovirales, Demerecviridae, Markadamsvirinae, Tequintavirus, Unclassified Tequintavirus |
